# Deep Digital Flexor Tendon Injury at the Level of the Proximal Phalanx in Frontlimbs With Tendon Sheath Distension Characterized by Standing Low-Field Magnetic Resonance Imaging in Horses: 13 Cases (2015–2021)

**DOI:** 10.3389/fvets.2021.734729

**Published:** 2021-11-18

**Authors:** Elisabeth Cornelia Susanna van Veggel, Kurt T. Selberg, Brenda van der Velde-Hoogelander, Katrien Vanderperren, Stefan Marc Cokelaere, Hendrik-Jan Bergman

**Affiliations:** ^1^Sporthorse Medical Diagnostic Centre, Heesch, Netherlands; ^2^Department of Environmental and Radiological Health Sciences, College of Veterinary Medicine and Biomedical Sciences, Colorado State University, Fort Collins, CO, United States; ^3^Department of Medical Imaging of Domestic Animals and Orthopedics of Small Animals, Faculty of Veterinary Medicine, Ghent University, Merelbeke, Belgium

**Keywords:** MRI, tendon sheath, DDFT, equine, tenoscopy

## Abstract

**Objective:** To describe the MRI findings for 13 horses with deep digital flexor tendon (DDFT) injury at the proximal phalanx where the tendon goes from ovoid to bilobed in frontlimbs with tendon sheath distension. In addition, the prognosis of this lesion was assessed.

**Design:** Retrospective case series.

**Animals:** Thirteen client-owned horses.

**Procedures:** Medical records were reviewed, and data were collected regarding signalment, history, MRI findings, and outcomes of horses. Findings of MRI were recorded and whether the case was confirmed with tenoscopy.

**Results:** A diagnosis of DDFT injury at the junction between ovoid and bilobed portions at the level of the proximal phalanx was established in 13/20 (65%) horses that underwent MRI examination of the frontlimb digital flexor tendon sheath. Return to previous level of work was poor in this subset of horses with only three of 13 (23%) horses returning to previous level of work and one horse still in rehabilitation.

**Conclusions and Clinical Relevance:** Standing low-field MRI represents a potentially useful diagnostic tool to evaluate digital flexor tendon sheath distension especially when evaluating the DDFT at the proximal phalanx where the tendon progresses from ovoid to bilobed. Prognosis of lesions of the DDFT at the proximal phalanx appears less favorable than previously reported causes of tendon sheath distension.

## Introduction

Digital flexor tendon sheath (DFTS) distension is a common condition seen among horses and may or may not be associated with lameness (1–7). Diagnostic imaging techniques can assist to find the underlying reason for tendon sheath distension by identifying injury of the internal structures. The DFTS is a synovial structure containing the superficial and deep digital flexor tendons as well as the manica flexoria, mesotendons, and synovial plicae, and it extends from the distal third of the metacarpus/metatarsus to the middle third of the second phalanx (8, 9). The DFTS helps to lubricate and stabilize the passage of the tendons through the highly mobile fetlock canal and the pastern region (10). There are three annular ligaments that the palmar wall of the sheath incorporates: the palmar annular ligament and the proximal and distal digital annular ligaments. The annular ligaments are thickening of the fibrous layer of the wall with a transverse fiber orientation measuring <2 mm (8). The manica flexoria (MF), the ring from the superficial digital flexor tendon (SDFT) that encircles the DDFT, is located proximal to the proximal sesamoid bones. A second ring at the level of the proximal phalanx is present and, together with MF, holds the DDFT in a proper position (9).

Distension of the DFTS and potential associated lameness have been attributed to tendinitis of the SDFT, tendinitis of the DDFT, desmitis of the PAL, DFTS rupture, longitudinal tears of the DDFT, complex tenosynovitis, desmitis of the proximal digital annular ligament (PDAL), tears of the MF, proliferative synovitis of unknown cause, and infectious tenosynovitis (1, 2, 4, 6, 8, 11–16).

The DFTS has historically been examined *via* tenoscopy, ultrasonography, and radiography including the use of negative and positive contrast (6, 10, 17). Ultrasonography has a sensitivity of 71% and specificity of 71% for detection of marginal tears of the DDFT (6). Contrast (positive) radiography is reported to have specificity of 73% and sensitivity of 54% for diagnosing DDFT tears (17). Investigation of the tendon sheath using computed tomographic tenography has also been described (18, 19). A recent cadaver study using a saline injection of the DFTS improves the visualization of the internal structures with MRI and ultrasonography (20).

The aim of this article is to describe specific tears of the DDFT at the junction between ovoid and bilobed portions within the proximal aspect of the proximal phalanx as seen on MRI and report their prognosis.

## Materials and Methods

### Case Selection

Medical records of horses that underwent standing low-field MRI for examination of their DFTS distension at the Sporthorse Medical Diagnostic Centre (SMDC) between September 1 2015, and January 1, 2021, were reviewed. The criteria for inclusion were availability of a clinical history, MRI examination of the entire tendon sheath, and follow-up information. Only patients that were sent for an MRI examination because of DFTS concerns were included. From this DFTS group, cases with a lesion of the DDFT at the junction between ovoid and bilobed portions at the level of the proximal phalanx were selected. MRI studies were evaluated by Dr. Kurt Selberg, (DVM, MS., DACVR) as well as Dr. Elisabeth van Veggel (DVM, ECVDI resident). Consensus on MRI findings was reached on all studies.

### Data Collection

Data collected from the records of each horse include age, breed, sex, work discipline and level, findings from diagnostic imaging, and outcome. Successful outcome was return to similar or higher level of work. Whether the horses had tenoscopic exploration was also recorded. Clinical and ultrasonographic examination was not performed on all cases and therefore not included as part of the study, as many were referral cases that were sent for an MRI examination only. Owner consent was not specifically obtained for this study as owners give their consent for research purposes as part of the registration process at SMDC.

### Magnetic Resonance Imaging

A standing low-field (0.27-T) MRI system was used for image acquisition (Hallmarq Veterinary Imaging, Guildford, UK). Transverse images for examination of the entire tendon sheath were collected from the foot up to the mid metacarpus using a minimum of T1W GRE, T2^*^W GRE, and T2W FSE images. In 11/13 cases, STIR FSE images were also acquired. The MRI parameters used in the various sequences are noted in Table 1. The dorsal margin of the DDFT was carefully examined for irregularities and altered signal intensity (SI). Furthermore, tissue extending from the dorsal border of the DDFT into the tendon sheath was also noted as well as any additional tendinous lesions.

**Table 1:** Standing low-field MRI parameters used during tendon sheath examination in this study.

**Sequence**	**TR (ms)**	**TE (ms)**	**Flip angle (deg.)**	**FOV (cm)**	**Matrix size**	**Slice thickness (mm)**	**Gap (mm)**	**FrxPh**
T1W GRE	52	8	50	170 × 170	256 × 256	5	1	170 × 170
T2*W GRE	68	13	28	170 × 170	256 × 256	5	1	170 × 160
T2W FSE	1,500	110	90	170 × 170	256 × 256	5	1	128 × 144
STIR FSE	1,834	22	90	170 × 170	256 × 256	5	1	128 × 144

*TR, repetition time; TE, echo time; FOV, field of view. FrxPh, number of frequency encoding steps and the number phase encoding steps*.

## Results

Between September 2015 and January 2021, 20 cases were referred to SMDC for MRI examination of their frontlimb DFTS distension. In this group of horses, 13 horses presented with DDFT injury at the level of the proximal phalanx at the junction between ovoid and bilobed portions. There were eight right front and five left front limbs. The 13 cases were represented by nine geldings, three mares, and one stallion. The mean age was 7 years and median 6 years (range 5–15 years). Seven out of 13 cases were of horses 5 or 6 years of age. A variety of disciplines were presented which included show jumping (three cases), dressage (seven cases), eventing (two cases), and recreational riding (one case). Five horses were competing and/or training at a low level or used for recreational riding, and eight horses were competing and/or training at a medium to high level. Horses had a unilateral 1–2/5 AAEP grade lameness at the time of their clinical examination but were not reexamined prior to the MRI.

Lesions were evident on all sequences in the majority of cases (11/13 cases). In two cases, lesions were more clearly represented on T2W FSE and STIR FSE transverse images. In one of these two cases, the lesion was evident on T2^*^W GRE transverse images as well but not on the T1W GRE images. All horses had moderate to severe distension of the tendon sheath. In 12/13 (92%) cases, the lesion was at the dorsolateral aspect of the DDFT. In the other case, the lesion was at the dorsomedial aspect of the DDFT.

**Figure 1:**
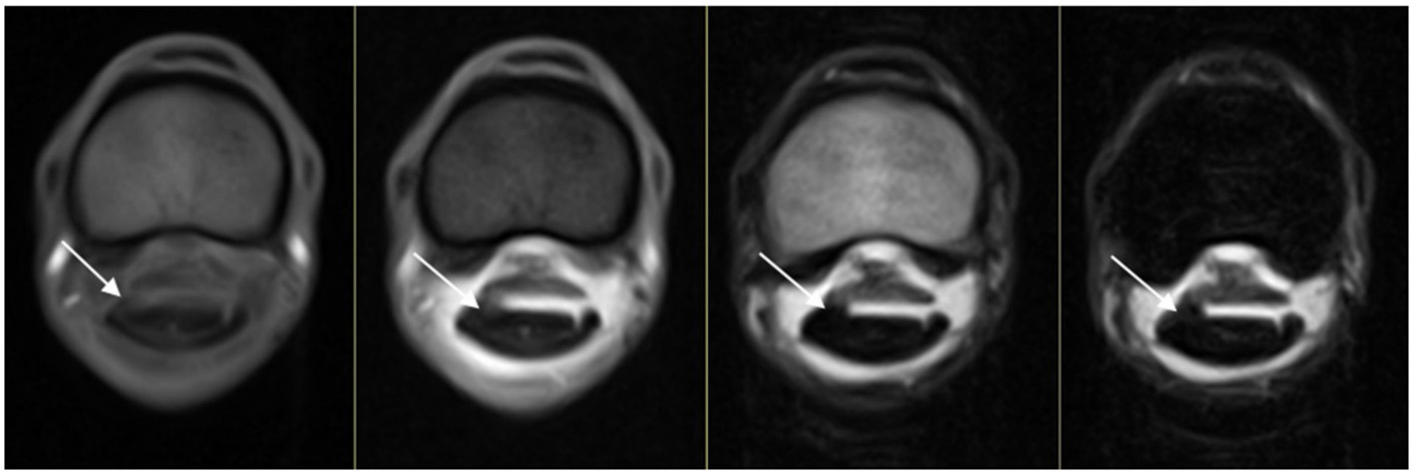
From left to right, T1WGRE, T2*W GRE, T2WFSE, and STIR FSE images of a dorsolateral DDFT injury at the level of the proximal phalanx where the DDFT goes from ovoid to bilobed in an RF limb (lateral is to the left). The injury is evident on all sequences (white arrow) and is of intermediate SI on T1WGRE and T2*WGRE and low SI on T2WFSE and STIR FSE images.

**Figure 2:**
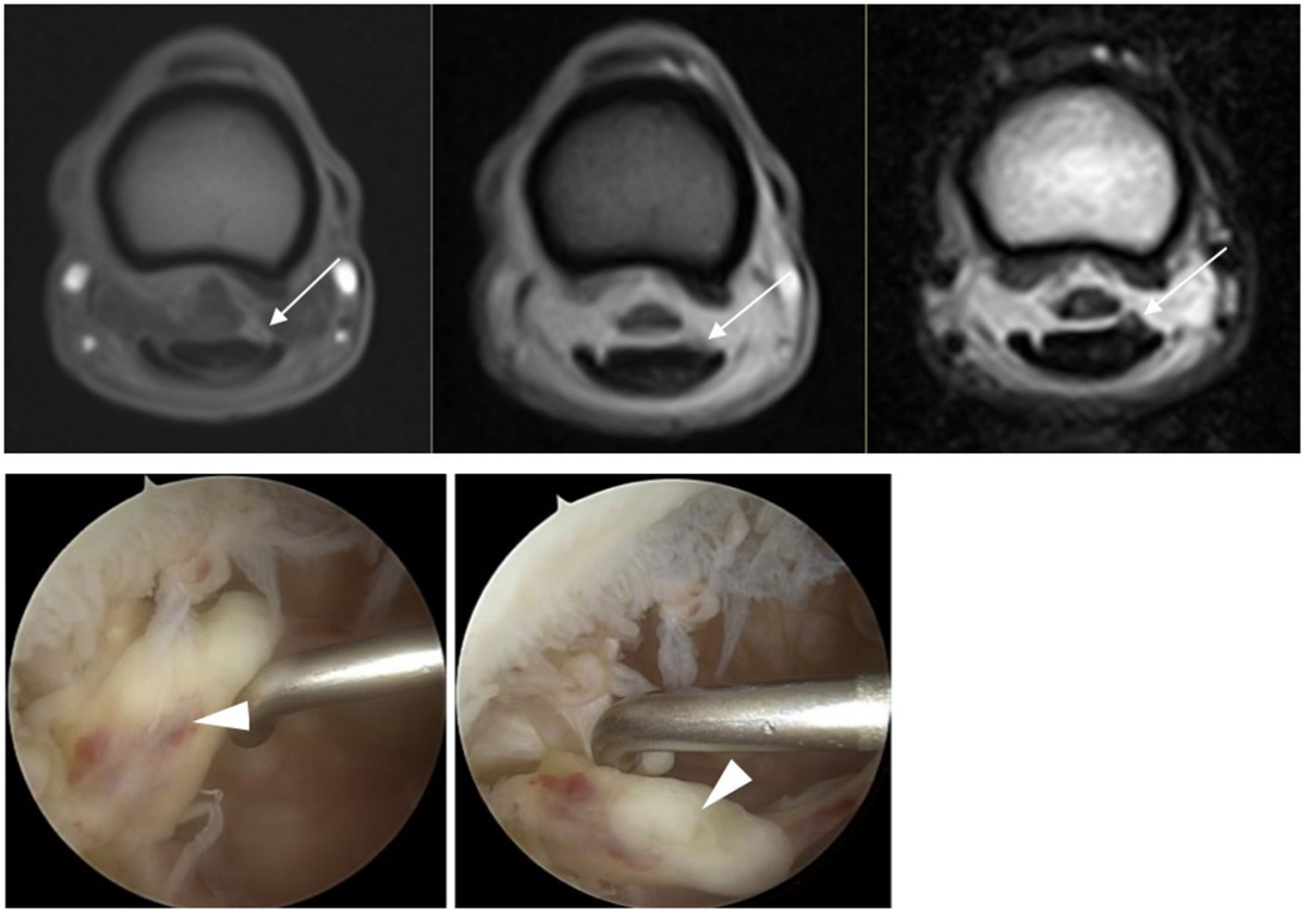
In the top row, from left to right T1W GRE, T2*W GRE, and T2W FSE(c) images of a dorsolateral DDFT injury at the level of the proximal phalanx where the DDFT goes from ovoid to bilobed in a LF limb (lateral to the right of the image). The injury is evident on all sequences (white arrow) and has high SI on T1W GRE and intermediate SI on T2*W GRE and T2W FSE images. The appearance during tenoscopic examination is shown in the bottom row images (white arrowhead) (tenoscopic images courtesy of Dierenkliniek Emmeloord).

**Figure 3:**
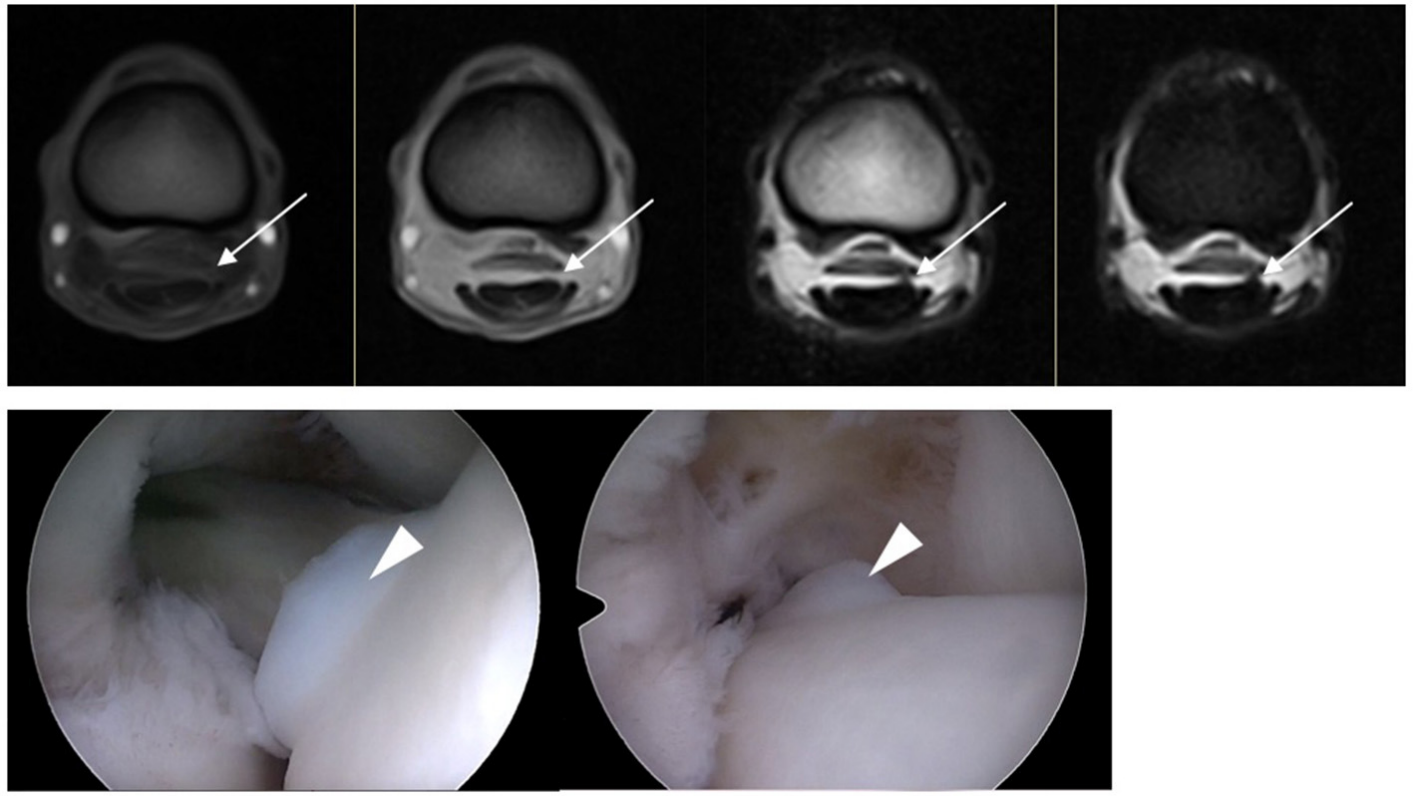
In the top row, from left to right T1W GRE, T2*W GRE, T2W FSE, and STIR FSE images of a dorsolateral DDFT injury at the level of the proximal phalanx where the DDFT goes from ovoid to bilobed in a LF limb (lateral to the right of the image). The injury is clearly evident with low to intermediate SI on T2W FSE and STIR FSE and less well visualized on T1W GRE and T2*W GRE (white arrow). The appearance during tenoscopic examination is shown in the bottom row images (white arrowhead) (tenoscopic images courtesy of *Via Nova*).

All cases had irregularity of the dorsal margin as well as altered signal intensity of the dorsal margin of the DDFT. The altered signal intensity of the dorsal margin was intermediate or high SI on T1W GRE images, intermediate SI on T2^*^W GRE images, and intermediate or low SI on T2WFSE and STIR FSE images. Irregular marginated tissue projecting from the dorsal border of the DDFT into the tendon sheath was noted in 11/13 cases. This proliferative tissue was of intermediate or high signal SI on T1W GRE, intermediate SI on T2^*^W GRE, and low or intermediate SI on T2W FSE and STIR FSE images in all cases. Figures 1, 2 demonstrate a dorsolateral injury evident on all acquired sequences. For the patient in Figure 2, no transverse STIR FSE was acquired. Figure 3 demonstrates a lesion that was evident on transverse T2W FSE and STIR FSE but not clearly seen on T1W GRE and T2^*^W GRE. The lesions in Figures 2, 3 were confirmed during tenoscopy.

As shown in Table 2, in seven patients the DDFT at the junction between the ovoid and bilobed portions was the only lesion. Six patients had an additional injury site associated with the tendon sheath. Of these, two patients had a thickening of the distal digital annular ligament (DDAL). The DDAL was thickened throughout in one case and thickened laterally prior to insertion onto the proximal phalanx in the other case. One patient had a focal SDFT injury laterally within the proximal pastern, and one patient had a focal SDFT injury proximolaterally to the proximal sesamoid bones. One patient had a dorsolateral DDFT injury proximal to the sesamoid bones, and one patient that had a diffusely thickened MF.

**Table 2:** Age, sex, use, limb, location of injuries, confirmation during surgery, and ability to return to work of the 13 horses.

**Case number**	**Sex**	**Age**	**Discipline**	**Limb**	**DDFT injury location**	**Additional injury within tendon sheath**	**Surgery**	**Return to work**
1	S	5	Recreational	RF	Dorsolateral	NO	NO	YES
2	G	6	Dressage	RF	Dorsolateral	DDAL	NO	NO
3	G	5	Eventing	RF	Dorsolateral	SDFT (lat)	NO	NO
4	G	5	Dressage	LF	Dorsolateral	DDFT (lat)	NO	NO
5	M	15	Show jumping	RF	Dorsomedial	DDAL (lat)	NO	NO
6	G	8	Show jumping	RF	Dorsolateral	NO	NO	NO
7	G	6	Eventing	RF	Dorsolateral	NO	NO	NO
8	G	6	Dressage	RF	Dorsolateral	SDFT (lat)	YES	YES
9	G	13	Dressage	LF	Dorsolateral	NO	YES	NO
10	G	9	Dressage	LF	Dorsolateral	NO	YES	REHAB
11	M	10	Dressage	LF	Dorsolateral	NO	YES	YES
12	G	5	Dressage	LF	Dorsolateral	NO	YES	NO
13	M	7	Show jumping	RF	Dorsolateral	MF (diffuse)	YES	NO

*G, gelding; M, mare; S, stallion*.

Six of 13 cases were confirmed with surgery, and in all cases the injured structure (DDFT) as well as location of the injury during surgery matched the MRI location of the injury considering the difference in limb loading with weight bearing vs. non-weight bearing. No additional lesions were identified during surgery. One horse's lesion was also confirmed postmortem. In the surgery group, there were two patients that had an additional MRI lesion that was also confirmed during surgery. These were the patients with additional MF thickening and SDFT injury proximal to the sesamoid bones.

In total, only three of 13 cases returned to competition at the same or higher level of work and one of 13 cases was still in rehabilitation. Out of the surgical patients, two out of six returned to previous or higher level of work, three out of six did not, and one out of six was still in rehabilitation. The two cases that returned to similar or higher level of work competed at the international level in dressage, and the one case that returned to similar or higher level of work with conservative treatment was used for recreational riding.

## Discussion

The present study describes findings for 13 horses with DDFT injury at the proximal phalanx where the tendon goes from ovoid to bilobed. Results of the present study provide an evaluation of the diagnostic value of standing low-field MRI for the detection of DDFT injury and the associated MRI appearance on various sequences. This study highlights an area which may be underestimated with other imaging modalities and where careful inspection should be made during scanning and interpretation of the MRI images. In this group of horses, the prognosis was poor with only three of 13 (23%) horses returning to previous level of work. At the time of the study, one horse was still in rehabilitation.

The slight variability of the lesions with the different sequences highlights the importance of performing all sequences listed (T1W GRE, T2^*^W GRE, T2W FSE, and STIR FSE). Most cases (11/13, 85%) were evident on all sequences. There were two cases where the lesion was not clearly seen on T1W GRE or T2^*^W GRE images. This may be related to inherent adjacent tissue contrast, motion, and slice positioning. Although all sequences were acquired if possible at the exact same location, patient movement in some cases forced the examiner to re-pilot which may have moved slice positioning.

All horses had moderate to marked tendon sheath distension. Accurate length of time of tendon sheath distension and injury were not available for all cases and therefore not included in this study, but it should be noted that this may affect the degree of the distension. In the normal tendon sheath, the amount of fluid present within the DFTS is variable and its synovial lining is smooth. The SDFT and DDFT have a homogenous low SI on MRI examination except at the level of the MCP/MTP joint itself where the tendons conform around the angle of the fetlock joint and there is often linear intermediate SI noticeable within the dorsal third of the DDFT (19). However, increased SI, altered shape, and size of the flexor tendons are considered abnormal findings (19). The tissue extending from the dorsal border of the DDFT is consistent with torn tissue, granulation tissue, and/or fibrous tissue, and in the subset of patients with surgery, this was confirmed on tenoscopic evaluation. Immature granulation tissue has high SI on T2W FSE while mature fibrotic tissue has low SI (21).

All patients in this article were referred for MRI because a diagnosis was not made using ultrasonography and/or doubt was present in the diagnosis. It must be kept in mind that the poor prognosis in this subset of patients may be due to the selection of patients sent for MRI. A sound horse with distension of the tendon sheath may not be referred for a MRI examination. Also, more complex and chronic cases may have been more likely to be referred for further imaging and so the current findings may be skewed or biased. As previously noted, marginal tears of the DDFT carry a worse prognosis than other lesions within the tendon sheath with 42% of horses returning to previous level of work in contrast to 67% in horses with MF tears (6). Only three of 13 (23%) of horses in our study returned to work which is significantly lower than that previously reported (5, 6). Interestingly, seven of 13 (53%) horses were 5 or 6 years of age and the mean and median age were lower than previously reported (4, 5).

MRI examination often results in finding multiple injuries and lesions that are commonly clustered to one side of the limb, suggesting that there may be a component of generalized trauma and/or overloading (19). Our study showed injury clustering in half the cases where additional lesions were present (three out of six), but our study population may be too small to conclude on this.

In our selection of 13 patients with injury to the DDFT at the specific region where the DDFT goes from ovoid to bilobed, there were only frontlimbs involved. Of the 25 cases in which the DFTS was scanned during this time, there were five hindlimbs. It was previously noted that significantly more frontlimb tendon sheaths were affected than hindlimbs and that the RF is significantly more affected than the LF in jumpers (14). This may be due to loading patterns and/or track design leading to increased injuries on the RF. All affected horses had been in regular work prior to the tendon sheath distension, and the onset of distension was reported to be acute in all cases. As previously reported, adherence of torn tendinous tissue to the DFTS wall or adjacent structures may have been possible (6). The predilection toward the lateral side has been previously reported and is possibly related to biomechanics. The junction between the ovoid and bilobed portions of the DDFT may provide a zone of localized stress, thereby predisposing this area to injury.

Although the number of cases that had surgery was low, there was a slightly better success after tenoscopic debridement than the non-surgical cases. Exposed torn collagenous tissue appears to be a source of irritation to the synovial membrane, and therefore tenoscopy is recommended to optimize healing (4, 5). Within a tendon sheath, there are no innate mechanisms available that can remove disrupted collagen fibers (4).

When appropriate, MRI and surgical techniques may complement each other as diagnostic and treatment modalities, respectively. Early intervention is recommended as preoperative marked distension may reflect the severity of synovial insult (6). All cases presented in this article had moderate to marked distension of the tendon sheath possibly reflecting the severity of the injury. Accurate diagnosis and directed treatment is vital in managing patients with DFTS distension. No cases showed signs of palmar annular constriction syndrome.

The main limitation was the low number of horses and the lack of horses that had tenoscopic findings with negative MRI findings. Another limitation was the absence of a repeatability study for the image interpretations by the two observers.

In conclusion, standing low-field MRI can be used to evaluate DFTS distension especially when evaluating the DDFT at the proximal phalanx where the tendon progresses from ovoid to bilobed. Prognosis of these lesions was guarded in this group of 13 horses.

## Data Availability Statement

The raw data supporting the conclusions of this article will be made available by the authors, without undue reservation.

## Ethics Statement

Ethical review and approval was not required for the animal study because the available data was part of a clinical examination. Written informed consent for participation was not obtained from the owners because all owners sign a waiver when registering their horses at the clinic, that the data can be used for research purposes.

## Author Contributions

All authors contributed to manuscript concept, preparation, and editing. All authors contributed to the articles and approved the submitted version.

## Conflict of Interest

The authors declare that the research was conducted in the absence of any commercial or financial relationships that could be construed as a potential conflict of interest.

## Publisher's Note

All claims expressed in this article are solely those of the authors and do not necessarily represent those of their affiliated organizations, or those of the publisher, the editors and the reviewers. Any product that may be evaluated in this article, or claim that may be made by its manufacturer, is not guaranteed or endorsed by the publisher.
